# Effect of Dietary Advanced Glycation End Products on Mouse Liver

**DOI:** 10.1371/journal.pone.0035143

**Published:** 2012-04-04

**Authors:** Raza Patel, Susan S. Baker, Wensheng Liu, Sonal Desai, Razan Alkhouri, Rafal Kozielski, Lucy Mastrandrea, Adil Sarfraz, Weijing Cai, Helen Vlassara, Mulchand S. Patel, Robert D. Baker, Lixin Zhu

**Affiliations:** 1 Department of Pediatrics, Digestive Diseases and Nutrition Center, State University of New York, Women and Children’s Hospital of Buffalo, Buffalo, New York, United States of America; 2 Department of Pathology, State University of New York, Women and Children’s Hospital of Buffalo, Buffalo, New York, United States of America; 3 Department of Pediatrics, State University of New York, Women and Children’s Hospital of Buffalo, Buffalo, New York, United States of America; 4 Division of Nephrology, Mount Sinai School of Medicine, New York, New York, United States of America; 5 Department of Biochemistry, State University of New York, Buffalo, New York, United States of America; University of Chicago, United States of America

## Abstract

The exact pathophysiology of non-alcoholic steatohepatitis (NASH) is not known. Previous studies suggest that dietary advanced glycation end products (AGEs) can cause oxidative stress in liver. We aim to study the effects of dietary AGEs on liver health and their possible role in the pathogenesis of NASH. METHODS: Two groups of mice were fed the same diet except the AGE content varied. One group was fed a high AGE diet and the second group was fed a regular AGE diet. Liver histology, alanine aminotransferase, aspartate aminotransferase, fasting glucose, fasting insulin, insulin resistance and glucose tolerance were assessed. RESULTS: Histology revealed that neutrophil infiltration occurred in the livers of the high AGE group at week 26; steatosis did not accompany liver inflammation. At week 39 livers from both groups exhibited macro- or micro-steatosis, yet no inflammation was detected. Higher insulin levels were detected in the regular AGE group at week 26 (P = 0.034), compared to the high AGE group. At week 39, the regular AGE group showed higher levels of alanine aminotransferase (P<0.01) and aspartate aminotransferase (P = 0.02) than those of the high AGE group. CONCLUSIONS: We demonstrate that a high AGE diet can cause liver inflammation in the absence of steatosis. Our results show that dietary AGEs could play a role in initiating liver inflammation contributing to the disease progression of NASH. Our observation that the inflammation caused by high AGE alone did not persist suggests interesting future directions to investigate how AGEs contribute to pro-oxidative and anti-oxidative pathways in the liver.

## Introduction

Nonalcoholic fatty liver disease (NAFLD) is the most common cause of abnormal liver enzymes in the U.S. [1], accounting for a quarter of all chronic liver diseases [2]. NAFLD is linked to excessive accumulation of triglyceride (TG) in the liver and encompasses a broad spectrum of liver pathology, ranging from steatosis to nonalcoholic steatohepatitis (NASH) with fibrosis that may progress to liver cirrhosis, portal hypertension, and even hepatocellular carcinoma. NAFLD occurs in approximately 40–90% of all obese individuals and 15% of those individuals progress to NAFLD’s severe form, NASH [3], [4], [5]. The incidence of NAFLD and NASH has been steadily increasing as the prevalence of obesity continues to rise [2]. Currently, NASH is the third most common indication for liver transplantation in the United States and is projected to become the most common reason for liver transplantation in the near future [6].

Evidence from animal models and human studies suggests that oxidative stress is one of the key factors in the disease progression of NASH [7], [8], [9]. Oxidative stress in the liver of NASH patients could arise from several pathways. One pathway is related to impaired electron flow through the mitochondrial respiratory chain (MRC) [10], [11]. When normal electron flow is interrupted, electrons are transferred to oxygen molecules from respiratory intermediates, generating superoxide anions and hydrogen peroxide [7], [12]. MRC impairment also increases fatty acid oxidation at peroxisomes and endoplasmic reticulum, resulting in the production of additional reactive oxygen species (ROS) [13], [14]. In addition, an animal model [15] and a human study [16] suggest that alcohol produced by obesity-related colonic microbes can contribute to the elevated ROS production.

Advanced glycation end products (AGEs) are potent trigger for ROS production in NASH livers [17]. AGEs are a heterogeneous group of molecules formed from the nonenzymatic reaction of reducing sugars with free amino groups of proteins, lipids and/or nucleic acids. AGEs may be formed exogenously by heating sugars with fats or proteins (Maillard reaction), from tobacco smoke, or endogenously through normal metabolism and aging [18], [19], [20]. *In vitro* experiments with a hepatic stellate cell line LI90 demonstrated that AGEs increase ROS production by NADPH oxidase and MRC [21]. This was confirmed by an *in vivo* study, in which mice fed a high AGE diet had lower levels of reduced glutathione/oxidized glutathione ratio and higher levels of 8-isoprostanes (lipid peroxidation products) than mice fed a low AGE diet [22]. To understand the effects of AGEs on liver and their possible role in the pathogenesis of NASH, we fed diets containing different amounts of AGEs to mice. Examination of the liver morphology and function revealed that a diet high in AGEs could induce inflammation independent of liver fat deposition.

## Materials and Methods

### Animals

All procedures involving mice were reviewed and approved by the Institutional Animal Care and Use Committee of University at Buffalo. Four week old C57BL/6NHsd male mice were purchased from Harlan Laboratories (Indianapolis, IN). These mice were individually caged and provided free access to water. Mice were divided into two groups: 1) regular AGE group, mice were fed with TestDiet 58G7 (TestDiet, Richmond, IN); 2) high AGE group, mice were fed with TestDiet 58G7 autoclaved at 120°C for 15 min, following a published method for the preparation of high AGE diet [23]. To monitor the growth of experimental groups, two mice fed *ad lib* with standard mouse chow (Teklad Global 18% Protein Rodent Diet, Harlan Laboratories) were used as reference animals. Fat contributed 12% of calories in TestDiet 58G7, compared to 18% in the standard mouse chow Teklad Global 18% Protein Rodent Diet. All mice in the study received the same calorie per gram body weight on a weekly basis.

### Measurement of AGEs

The concentrations of the major types of AGEs, carboxymethyl lysine and methylglyoxal, in mouse diets were determined by enzyme-linked immunosorbent assays as described previously [23].

### Histopathology

Five mice from each group were sacrificed on weeks 13, 26 and 39. Whole body perfusion was performed via left ventricle with oxygenated phosphate buffered saline supplemented with 1mM CaCl_2_ and 1mM MgCl_2_. Liver pieces of ∼5mm in all dimensions were taken from the mice and fixed in 4% formaldehyde for 15 min. The liver pieces were then sequentially equilibrated in 30% sucrose, 15% sucrose/50% OCT (Optimal Cutting Temperature medium, Sakura Finetek, Torrance, CA), and 100% OCT. The liver pieces were frozen in OCT before 10 µm sections were cut with a cryostat. The sections were stained with hematoxylin/eosin (H&E) or oil red O and visualized by light microscopy. A pathologist (R. K.) assessed each slide for steatosis, inflammation and fibrosis.

### Glucose, Insulin, Alanine Aminotransferase (ALT) and Aspartate Aminotransferase (AST) Measurements

Animals were fasted overnight before blood was collected via the submandibular vein with a sterile 5 mm point golden rod lancet (Medipoint, Mineola, NY). Glucose levels were measured using a FreeStyle Lite glucometer (Abbott Laboratories, Abbott Park, IL). Each reported value was the average of two readings. The remaining blood samples were incubated at 37°C for 40 min followed by centrifugation at 15,000g for 20 min at room temperature. Collected serum was aliquoted and stored at −80°C before further analysis. Insulin levels were determined with ELISA kits purchased from Mercodia (Uppsala, Sweden) following manufacturer’s instructions. Insulin resistance (IR) was calculated using the homeostasis model assessment (HOMA) method (fasting blood glucose (mmol/L) x fasting serum insulin (mU/L)/22.5 [24]. Serum ALT and AST activities were measured with ALT Enzymatic Assay Kits and AST Enzymatic Assay Kits (Bioo Scientific, Austin, TX) following product manuals.

### Glucose Tolerance Test

Intraperitoneal glucose tolerance tests (IPGTT) were performed as described previously [25]. Briefly, glucose (2 mg/g body weight) was delivered intraperitoneally to each animal after an overnight fast. Glucose readings were taken from the tail immediately before and several times after glucose injection as specified in Figure legend.

### Statistics

All analysis for statistically significant differences was performed with Student's t test with a two-tailed distribution. P values ≤0.05 were considered significant.

## Results

### AGEs and Animals

To evaluate the effect of dietary AGE on mouse liver, a high AGE mouse diet was prepared by high temperature treatment. The AGE contents in the diets were measured by ELISAs with antibodies against two major types of AGE molecules, carboxymethyl lysine and methylglyoxal, respectively (Table 1). These measurements indicated that the high AGE diet had an AGE content about three times that of the regular AGE diet.

**Table 1:** AGE content in mouse diets.

Mouse chow	CML^a^ (U/g)	MG^b^ (nmol/g)
TestDiet 58G7	60649±1147^c^	1107±39
High-heat treated TestDiet 58G7	197305±17441	3899±91

a Carboxymethyl Lysine.

b Methylglyoxal.

c Sample mean±standard deviation.

**Figure 1:**
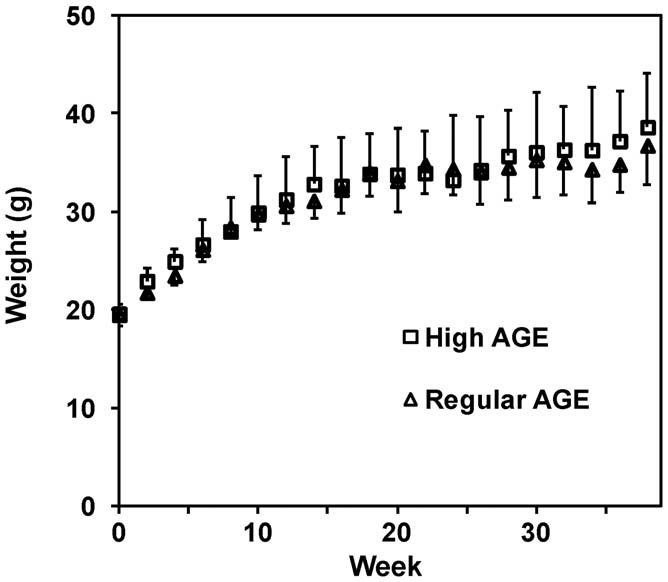
Growth curves. Body weights of mice fed with a high AGE diet or a regular AGE diet were plotted. Error bars represent standard deviations with plus direction for the high AGE group and the minus direction for the regular AGE group.

Two groups of mice were fed either a high AGE diet or a regular AGE diet. These two groups showed similar growth curves over the 39 weeks of the study; no significant difference was observed at any time point throughout the study (Figure 1). The growth curves of these two groups were also similar to that of the reference animals fed with regular mouse chow (data not shown).

### Histology

**Figure 2:**
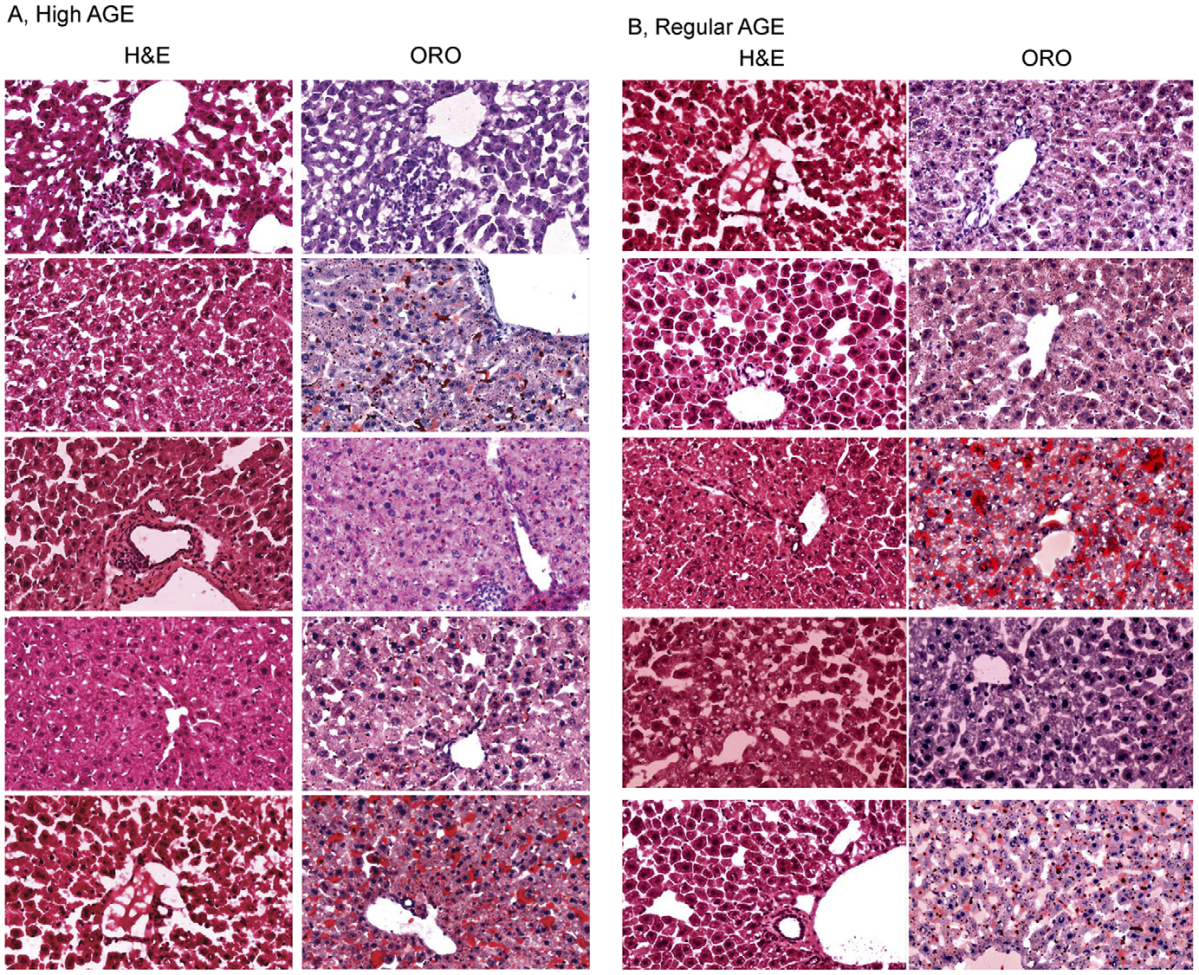
Liver histology of mice fed with a high AGE diet or a regular AGE diet for 26 weeks. Mice on the high AGE diet (A) or the regular AGE diet (B) for 26 weeks were whole-body perfused before liver collection. Cryosections of the livers were stained with H&E, or oil red O (ORO), respectively. Each row has the typical images of H&E and ORO staining of the same liver.

**Figure 3:**
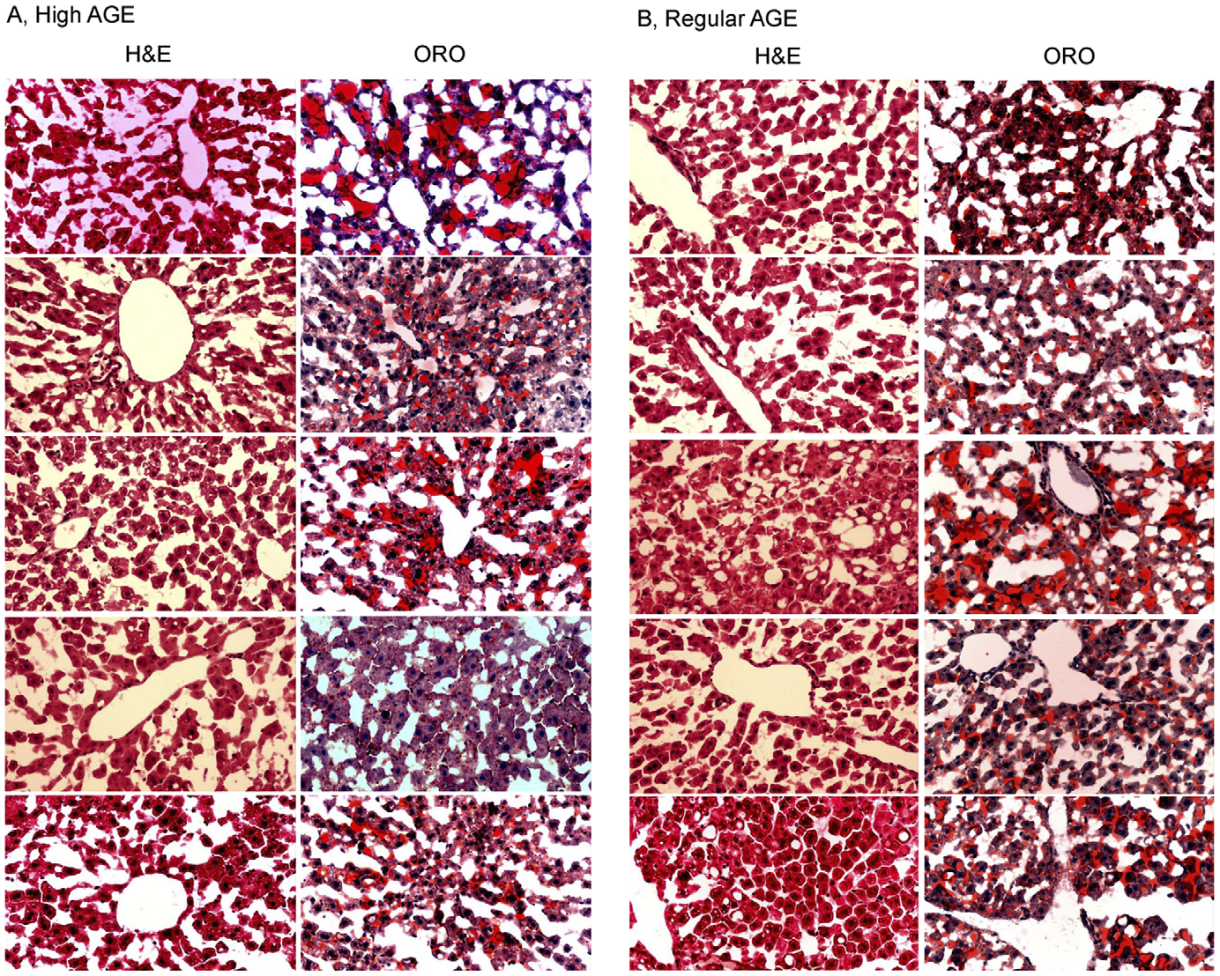
Liver histology of mice fed with a high AGE diet or a regular AGE diet for 39 weeks. Mice on high AGE diet (A) or regular AGE diet (B) for 39 weeks were whole-body perfused before liver collection. Cryosections of the livers were stained with H&E, or oil red O (ORO), respectively. Each row has the typical images of H&E and ORO staining of the same liver.

Five animals from each group were sacrificed at week 13, 26, and 39 to examine the possible effect of AGE on mouse livers. Cryosections of liver from each animal were stained with H&E and oil red O. Examination of the liver histology of all animals from the high AGE group and the regular AGE group showed no evidence of steatosis or inflammation at week 13 (data not shown). However, abnormalities were detected at week 26. Liver sections from two of the five high AGE-fed mice exhibited extensive macro- or micro-vesicular steatosis as indicated by oil red O staining (Figure 2A). More importantly, inflammatory foci were evident in the liver sections of two mice in the high AGE group. The inflammation observed was mild; no evidence of liver damage, regeneration or fibrosis was observed. It is noteworthy that livers with steatosis did not have inflammatory foci, while inflamed livers did not exhibit steatosis. Two out of the five mice from the regular AGE fed group exhibited steatosis at week 26, but no inflammatory focus was observed in the livers of this group (Figure 2B). It is striking that all liver sections from both groups exhibited extensive macro- or micro-vesicular steatosis at week 39 (Figure 3A, B), despite the fact that both diets are of low fat content (contributing 12% calorie). No inflammatory focus was observed in any section from either group at week 39 (Figure 3A, B).

### Liver Function Tests

**Figure 4:**
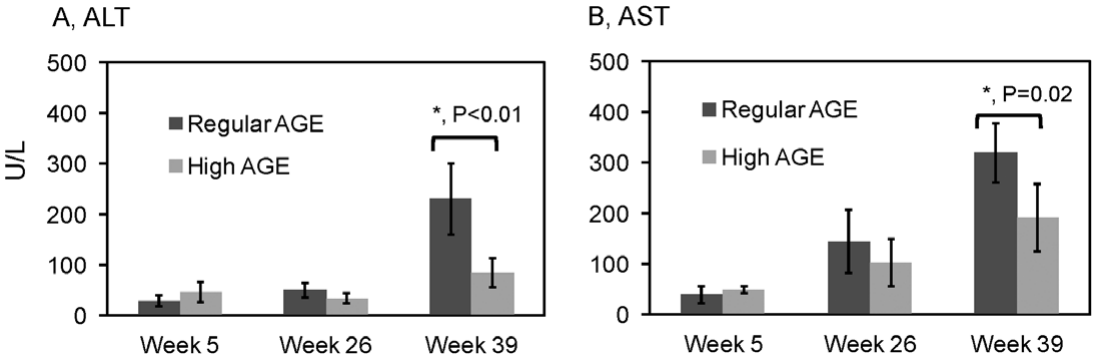
Measurement of serum ALT and AST. Sera were obtained from mice fed with a high AGE diet or a regular AGE diet. ALT (A) and AST (B) enzymatic activities were measured as specified in Materials and Methods. Error bars represent standard deviations (n = 5 for both groups).

Serum ALT and AST levels were measured to evaluate hepatocellular damage. Normal ALT and AST values were observed in all animals at week 5 of dietary treatment (Figure 4A & B). At week 26, the ALT levels remained normal in all animals (Figure 4A); however, marked elevation of AST levels were observed in both groups (Figure 4B). At week 39, ALT and AST levels from both groups were elevated (Figure 3A & B). Both ALT and AST were significantly higher in the regular AGE group compared to the high AGE group (p<0.01 and p = 0.02, respectively). These results indicate that liver injury occurred by 39 weeks in these animals and was more pronounced in those fed the regular AGE diet.

### Glucose Metabolism

**Figure 5:**
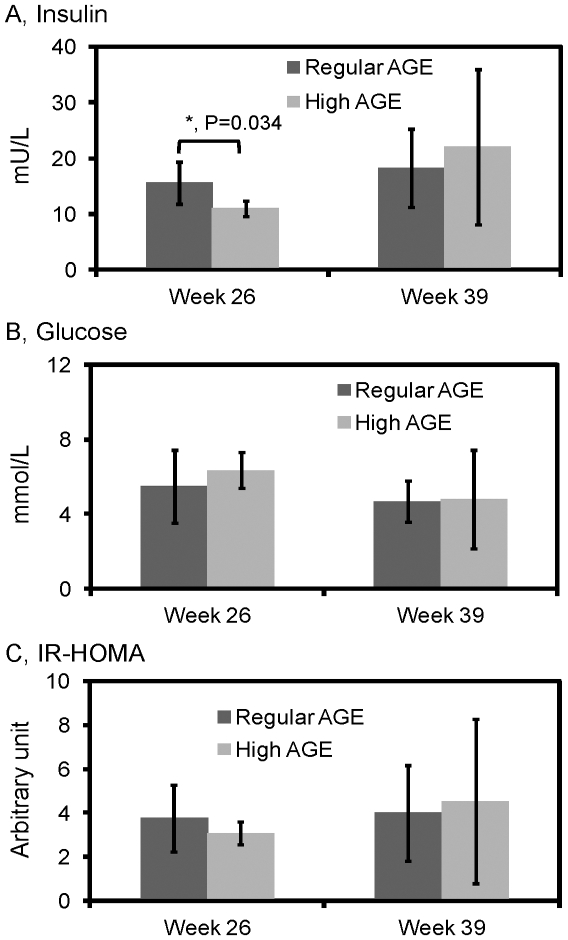
Intraperetoneal glucose tolerance test (IPGTT). Mice fed with a high AGE diet (n = 3) or a regular AGE diet (n = 4) were subjected to IPGTT. Blood glucose levels were measured immediately before (time = 0 min) and after (time = 15, 30, 60, 120 min) an IP injection of glucose (2 mg per gram body weight). Error bars represent standard deviations with plus direction for the high AGE group and the minus direction for the regular AGE group.

Because fat deposition in parenchymal cells (Figure 3 A & B) can contribute to abnormal metabolism, fasting insulin, glucose and insulin resistance in both groups were examined. Fasting insulin levels were higher in the regular AGE group compared to the high AGE group (P = 0.034) at week 26 (Figure 5 A). However, fasting glucose and HOMA-IR were comparable between the two groups (Figure 5B & C). At week 39, no significant difference was observed for fasting insulin, glucose or IR-HOMA between mice fed with different amounts of dietary AGE (Figure 5 A, B and C).

**Figure 6:**
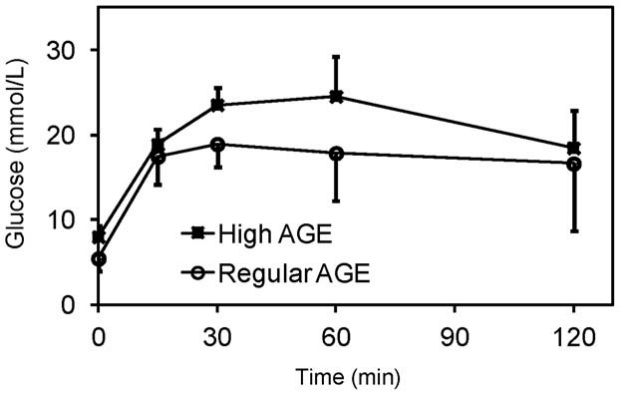
Measurement of fasting serum insulin, fasting blood glucose and insulin resistance. Mice fed with a high AGE diet (n = 5) or a regular AGE diet (n = 5) for 26 and 39 weeks were analyzed. A, serum insulin levels were determined by an ELISA kit as specified in Materials and Methods; B, blood glucose was measured with a glucometer; C, insulin resistance was evaluated according to the “homeostasis model assessment (HOMA)” method [24]: IR-HOMA  =  fasting glucose (mmol/L) x fasting insulin (mU/L)/22.5. Error bars represent standard deviations.

To further examine glucose homeostasis, intraperitoneal glucose tolerance tests (IPGTT) were performed at week 39. All animals exhibited glucose intolerance as evidenced by persistent hyperglycemia 2 h after intraperitoneal glucose injection (Figure 6). No significant difference in glucose was observed between the high AGE fed mouse group and the regular AGE fed mouse group at each time point tested (15 min, 30 min, 1 h and 2 h).

## Discussion

Most fatty livers do not develop liver inflammation [26], a hallmark of NASH. Factors contributing to the development of liver inflammation remain undefined. Our results show that dietary AGEs can cause liver inflammation and therefore contribute to the pathogenesis of NASH. Inflammatory foci were detected in mouse livers at week 26 when fed with a high AGE diet, while mice fed the regular AGE diet remained free from liver inflammation throughout the length of the 39 week study. This is consistent with the well-established concept that dietary AGE can initiate oxidative stress via AGE receptors expressed in liver cells [21], [22]. The fact that steatosis was not a characteristic of these inflammed livers suggests that dietary AGE alone is sufficient to cause liver inflammation, independent of steatosis. Therefore, our data suggest that dietary AGEs can play a role in initiating inflammation contributing to the disease progression of NASH. Our results are of immediate practical value for current and prospective NASH patients as AGEs are often major components of processed food [27], and one study demonstrated that serum AGE levels in NASH patients were higher than those of patients with steatosis and healthy controls [28].

On the other hand, some mice at week 26 and all animals at week 39 developed macro- or micro-vesicular steatosis, and all animals exhibited glucose intolerance at week 39, but there was no progression to liver inflammation with these fatty livers. These results recall the observation that ob/ob mice develop steatosis and insulin resistance [29] but not liver inflammation [30]. Our animal studies mirror the epidemiology of human NASH in that most people who have steatosis do not show progression to liver inflammation [26]. However, we were surprised that no inflammation was detected in any of the fatty livers in the high AGE fed group at week 39. It is broadly believed that steatosis sensitizes the liver to secondary insults in the development of liver inflammation and further liver complications [31]. As an agent to induce oxidative stress, dietary AGE could be one of the secondary insults. Yet, mild inflammation was detected only at week 26 but not at week 39 in the high AGE fed mice indicating that mild inflammation could resolve over time despite the appearance of steatosis in the presence of continued consumption of a high AGE diet. One possible explanation is that anti-oxidative mechanisms were activated to fight against the stress generated by the high AGE diet. Although AGEs induce oxidative stress via the cognate receptor RAGE, one study suggested that AGEs also induce glutathione S-transferase activity to function as an antioxidant [32]. This also provides a reasonable explanation for our observation that mice fed a high AGE diet exhibited significantly lower serum ALT and AST activities at week 39 than those on a regular AGE diet. We speculate that mechanisms may exist to silence the RAGE mediated pathway while maintaining the beneficial effects of AGE. We currently are examining known anti-oxidative pathways in these animals with the goal of identifying the mechanism(s) that protect the animals from oxidative stress. Our approach may reveal the molecular mechanism for how AGEs activate pro-oxidative but not anti-oxidative reactions.

Another possible explanation for this self-resolving inflammation is the low fat content in the diets of our study. One outstanding observation in our study was that fasting glucose, insulin, IR-HOMA and glucose tolerance were similar between high AGE and regular AGE fed mouse groups. This is in contrast to the observation of Sandu and colleagues [25] , who reported that a high AGE diet caused a ∼ 5 fold increase in fasting insulin level and significant glucose intolerance as compared to regular AGE fed animals. Sando *et al.* also found that plasma 8-isoprostane, a measurement of systemic oxidant stress, were significantly elevated in the high AGE diet fed animals. One major difference between our experimental approaches is that high fat diets were used in the study of Sandu *et al.*, while low fat diets were used in our study. Therefore, we speculate that dietary fat content differences contribute to the conflicting results. Perhaps, high AGE diet exacerbates the metabolic defect precipitated by a high fat diet. As such, it is of great interest to test whether low fat diets protect hepatocytes from the adverse effects of AGEs and prevented persistent liver inflammation. Another important question stemming from this discrepancy is whether a high AGE diet facilitates the development of liver inflammation on the background of a high fat diet and/or obese mouse model (db/db or ob/ob).

AGEs have been implicated in the pathogenesis of various diseases including diabetes [33], atherosclerosis [34], Alzheimer’s disease [35], and melanoma growth and metastasis [36], [37]. Vlassara and colleagues reported that a high AGE diet induces pro-inflammatory cytokines in diabetic patients [38], but ethical considerations make it impossible to obtain direct evidence that human liver inflammation is induced by a high AGE diet. Because we show neutrophil infiltration, our mouse study has unique value in supporting a causative link between AGE and liver inflammation. Chuyen and colleagues examined the effect of dietary AGE on the liver enzymes superoxide dismutase and glutathione-S-transferase in a rat model [39]. They reported no adverse effect of AGEs. The discrepancy between their study and ours may be explained by the fact that their rat study only lasted for 11 weeks and the authors did not examine the liver histology.

One of the striking and important aspects of our study is its length. For example, if this study had been terminated after a short period of time, as other studies of diet and liver injury have been, our observation that resolution of inflammation is possible would have been missed. This observation might lead to the understanding of mechanisms that could be used to mitigate harmful effects of diet on the liver.

In this study, liver inflammation occurred before steatosis in the high AGE diet group. This phenomenon may add to the debate on whether steatosis is a consequence of liver inflammation or vice versa (reviewed in [40]). Although similar levels of steatosis were observed between high and low AGE diet groups at week 39, cell type specific differences might have existed between two groups at some time points during the length of this study. Further investigation on individual liver cell types may reveal possible link between inflammation and steatosis and other aspects of the NASH pathophysiology.

In summary, we demonstrate that a high AGE diet causes liver inflammation in the absence of steatosis. This finding is relevant to patients at high risk for NASH and other liver injuries. Our observation that the inflammation caused by high dietary AGE alone did not persist suggests interesting future directions to investigate how AGE induced pro-oxidative pathways are regulated and how they may be down-regulated by AGE-induced anti-oxidative pathway. The animal model we developed is a valuable platform on which a therapeutic and preventive agent against the adverse effects of AGEs could be identified and tested.
